# Genome-wide profiling of long non-coding RNA of the rice blast fungus *Magnaporthe oryzae* during infection

**DOI:** 10.1186/s12864-022-08380-4

**Published:** 2022-02-15

**Authors:** Gobong Choi, Jongbum Jeon, Hyunjun Lee, Shenxian Zhou, Yong-Hwan Lee

**Affiliations:** 1 Interdisciplinary Program in Agricultural Genomics, Seoul National University, Seoul, 08826 Korea; 2 Plant Immunity Research Center, Seoul National University, Seoul, 08826 Korea; 3 Korean Bioinformation Center, Korea Research Institute of Bioscience and Biotechnology, Daejeon, 34141 Korea; 4 Department of Agricultural Biotechnology, Seoul National University, Seoul, 08826 Korea; 5 Center for Plant Microbiome Research, Center for Fungal Genetic Resources, Plant Genomics and Breeding Institute, and Research Institute of Agriculture and Life Sciences, Seoul National University, Seoul, 08826 Korea

**Keywords:** Host infection, Long non-coding RNA, *Magnaporthe oryzae*, RNA-seq, Transcriptional regulation

## Abstract

**Background:**

Long non-coding RNAs (lncRNAs) play essential roles in developmental processes and disease development at the transcriptional and post-transcriptional levels across diverse taxa. However, only few studies have profiled fungal lncRNAs in a genome-wide manner during host infection.

**Results:**

Infection-associated lncRNAs were identified using lncRNA profiling over six stages of host infection (e.g., vegetative growth, pre-penetration, biotrophic, and necrotrophic stages) in the model pathogenic fungus, *Magnaporthe oryzae*. We identified 2,601 novel lncRNAs, including 1,286 antisense lncRNAs and 980 intergenic lncRNAs. Among the identified lncRNAs, 755 were expressed in a stage-specific manner and 560 were infection-specifically expressed lncRNAs (ISELs). To decipher the potential roles of lncRNAs during infection, we identified 365 protein-coding genes that were associated with 214 ISELs. Analysis of the predicted functions of these associated genes suggested that lncRNAs regulate pathogenesis-related genes, including xylanases and effectors.

**Conclusions:**

The ISELs and their associated genes provide a comprehensive view of lncRNAs during fungal pathogen-plant interactions. This study expands new insights into the role of lncRNAs in the rice blast fungus, as well as other plant pathogenic fungi.

**Supplementary Information:**

The online version contains supplementary material available at 10.1186/s12864-022-08380-4.

## Background

Genomes encode large numbers of non-coding transcripts, which function in gene regulation [1–3]. Non-coding RNAs longer than 200 nucleotides are considered long non-coding RNAs (lncRNAs), in contrast to small non-coding RNAs, such as microRNAs and small interfering RNAs [4, 5]. Based on their genomic positions and contexts within protein-coding genes, lncRNAs are categorized as intergenic lncRNAs, antisense lncRNAs, sense lncRNAs, and intronic lncRNAs [5–7]. LncRNAs can also be classified as cis-acting lncRNAs, which regulate target genes at adjacent regions, and trans-acting lncRNAs, which function at independent chromosomal loci [8]. LncRNAs modulate the transcriptome through multiple dimensions, including epigenetic, transcriptional, post-transcriptional, translational, and post-translational levels [9].

Following the discovery of *H19* in humans and *Xist* in mice, many more lncRNAs have been functionally characterized [10, 11]. Several studies have reported that mammalian lncRNAs are associated with cell differentiation and disease process; they also serve as biomarkers for cancer diagnoses [12–14]. Plant lncRNAs, such as *COLDAIR* and *GhlncNAT*-*ANX2,* have roles in development and in defense against pathogens [15, 16].

Functional analysis of lncRNAs in fungi has mainly been carried out in the yeast species, *Saccharomyces cerevisiae* and *Schizosaccharomyces pombe*. Yeast lncRNAs modulate vegetative growth, sexual reproduction, cell–cell adhesion, and phosphate regulation [17, 18]. LncRNAs also regulate the circadian clock (*qrf*) and cellulase genes (*HAX1*) in the saprotrophic fungi *Neurospora crassa* and *Trichoderma reesei*, respectively [19–21]. LncRNA *RZE1* regulates zinc finger transcription factor *ZNF2* and affects the yeast-to-hypha transition in the human pathogenic fungus *Cryptococcus neoformans* [22]. LncRNAs have also been reported to play roles in vegetative growth (*ncRNA1*), metabolic processes (*carP*), asexual/sexual reproduction (*GzmetE-AS*), and pathogenicity (*as-um02151*) in plant pathogenic fungi [23–26]. While genome-wide profiling of lncRNAs has been performed in some fungi during vegetative growth and sexual development, the profiling of lncRNAs associated with the infection process of plant pathogenic fungi is generally incomplete and has only been studied in the rice smut fungus *Ustilaginoidea virens* [27–30].

Rice blast disease is caused by the filamentous fungus *Magnaporthe oryzae*, which is responsible for an annual yield loss of 10** − **30% [31]. In addition to its economic importance, this fungus has served as a model of host–pathogen interactions [32]. *M. oryzae* undergoes morphological and functional transitions during vegetative growth, appressorium formation, the biotrophic stage, and the necrotrophic stage during the infection process [33]. Following the completion of whole genome sequencing of this fungus, transcriptome profiling was performed to understand gene regulation during the infection process [34–37]. However, functional and genome-wide lncRNA investigations have not been performed in *M. oryzae*.

Here, we report the genome-wide identification of lncRNAs during specific stages of infection, including vegetative growth, pre-penetration, the biotrophic stage, and the necrotrophic stage. We identified infection-specifically expressed lncRNAs (ISELs), predicted the target genes using two different methods, and predicted the functions of ISEL-associated genes. This study expands the transcriptome-level knowledge of *M*. *oryzae,* from protein-coding genes to long non-coding transcripts; it also provides a novel foundation for understanding the role of non-coding RNAs in host–pathogen interactions.

## Results

### Genome-wide identification of lncRNAs in *M. oryzae*

**Fig. 1:**
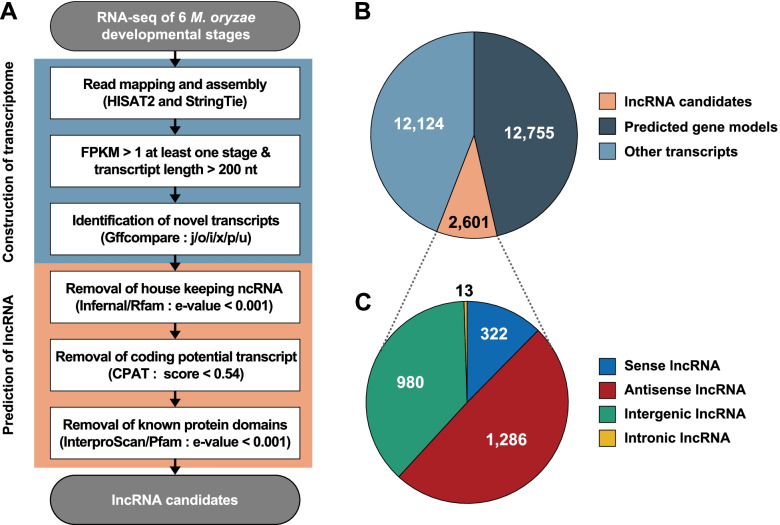
Schematic pipeline for identification of lncRNAs in *M*. *oryzae*. **A** Bioinformatic pipeline for lncRNA identification using RNA-seq data. CPAT, Coding Potential Assessment Tool. **B** Number of predicted transcripts. **C** Number of lncRNAs by different classes

RNA-seq data sets from vegetative mycelia, pre-penetration, biotrophic, and necrotrophic stages were used to identify lncRNAs during mycelial growth and disease development in *M. oryzae* [37]. Previously established pipelines were used to detect lncRNAs with some modifications (Fig. 1A) [38]. In total, 436.6 million reads were mapped to the *M*. *oryzae* genome with 27,480 predicted transcripts originating from 16,093 genomic loci (Fig. 1B). Among these transcripts, 23,586 transcripts were detected with an FPKM > 1 in at least one developmental or infection stage and were retained for further analysis. Novel transcripts (13,978) were identified using Gffcompare categorization [41]; known mRNAs from the Ensembl database and non-coding RNAs from the Rfam database were removed [42]. Coding transcripts were filtered out by removing coding potentials of < 0.54 and the remaining transcripts were scanned by InterProScan to remove transcripts carrying known protein domains. The resulting 2,601 lncRNA candidates were identified with a majority of antisense lncRNAs (1,286; 49.4%), intergenic lncRNAs (980; 37.7%), sense lncRNAs (322; 12.4%), and intronic lncRNAs (13; 0.5%) (Table 1, Fig. 1C). Of the identified 2,601 lncRNAs, 1,599 (61.5%) lncRNAs were expressed at all stages; 2,199, 2,183, 2,025, 2,075, 2,170, and 2,352 lncRNAs were expressed at the vegetative mycelia, 18 h post-inoculation (hpi), 27 hpi, 36 hpi, 45 hpi, and 72 hpi stages, respectively (Additional file 1: Fig. S1).

**Table 1:** Classification of lncRNAs in *M*. *oryzae*

Class of transcripts	Number of novel transcripts	Number of lncRNAs
Sense transcript	8,444	322
Antisense transcript	2,636	1,286
Intergenic transcript	2,876	980
Intronic transcript	22	13

### Genomic features of *M. oryzae* lncRNAs

**Fig. 2:**
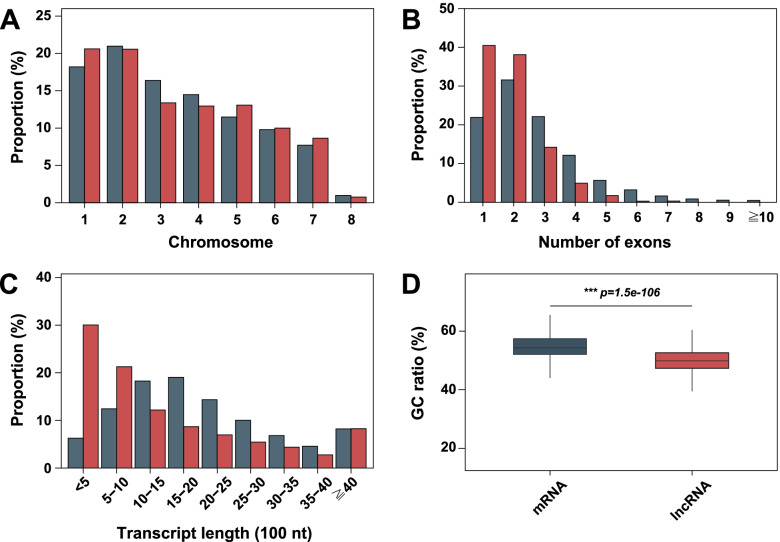
Genomic features of *M*. *oryzae* lncRNAs. **A** Distributions of mRNAs (bluish green) and lncRNAs (red) across chromosomes. **B** Distribution of transcript lengths. **C** Distribution of exon numbers per transcript. **D** GC ratio (%). The Wilcoxon–Mann–Whitney test confirmed a significant difference in GC ratio between the two groups. *** *p* < 0.001

Properties such as genomic distribution, exon number, length, and GC ratio of lncRNAs were investigated by mRNA comparisons. LncRNAs and mRNAs were differentially distributed across chromosomes (chi-squared test: *p* = 0.01413, test for equality of proportions: *p* = 5.635e-09) (Fig. 2A); lncRNAs (mean length = 1,584 nt) had shorter full-length transcripts than did mRNAs (mean length = 2,108 nt) (Fig. 2B). LncRNAs had fewer exons than did mRNAs (Fig. 2C); a greater proportion of lncRNAs possessed one or two exons, and lncRNAs exhibited a narrower range of exon numbers. The GC ratio of lncRNA (50.1%) was lower than the GC ratio of mRNA (55.5%) (Wilcoxon–Mann–Whitney test: *p* = 1.51153e-106) (Fig. 2D).

Conservation of *M. oryzae* lncRNAs was assessed by comparison to known lncRNAs from RNAcentral [46]. No significantly conserved lncRNA was discovered. We also compared lncRNA and mRNA sequences with genomic sequences from eight Magnaporthales species, along with *N*. *crassa* as an outgroup. *M*. *oryzae* lncRNAs were less conserved than mRNAs in all species; fewer than 10% of *M*. *oryzae* lncRNAs were conserved in most species, with the exception of *M*. *grisea* (Additional file 2: Fig. S2).

### Expression of lncRNA transcripts during infection

**Fig. 3:**
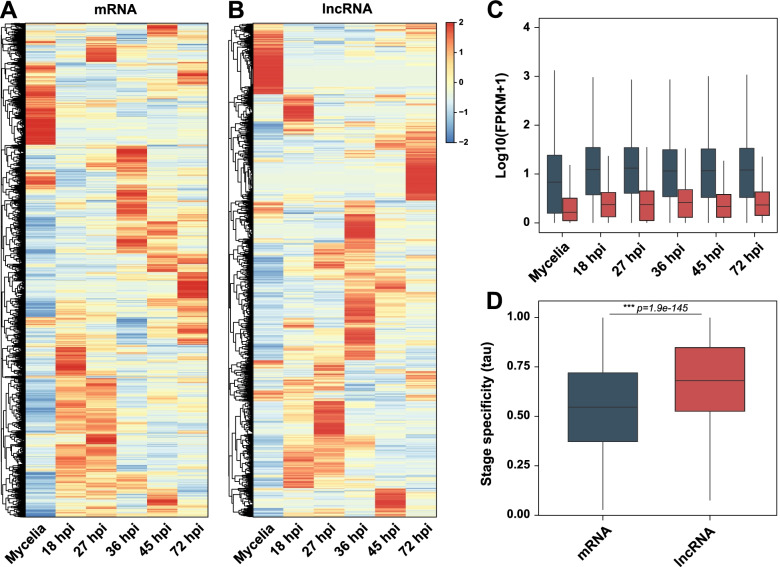
LncRNA expression level and pattern. **A**, **B** Expression heatmaps of 9,410 mRNAs and 2,601 lncRNAs, respectively. Z-score normalization was applied to FPKM values across stages. **C** Boxplot of mRNA (bluish green) and lncRNA (red) expression patterns across developmental and infection stages. **D** Density plot of transcript stage specificity over six stages. τ (Tau) is used as a stage specificity index. The index varies from 0 (consistently expressed transcripts) to 1 (perfectly stage-specific transcripts). The Wilcoxon–Mann–Whitney test confirmed a significant difference in tau distribution between the two groups. *** *p* < 0.001

The expression dynamics of lncRNAs were assessed by generating heatmaps based on FPKM values from the 9,410 detected mRNAs and 2,601 lncRNAs (Fig. 3A, 3B). Clustered, stage-specific expression patterns were identified for both mRNAs and lncRNAs. Mean FPKM values indicated that expression levels of lncRNAs (4.3–7.3) were much lower than expression levels of mRNAs (35.3–47.1) at the vegetative stage and all infection stages (Fig. 3C). LncRNAs showed the highest mean expression level at 45 hpi (7.3), whereas mRNAs showed the highest mean expression level at 18 hpi (47.1). We found that lncRNAs had higher expression levels in the infection stages, compared with the vegetative growth stage, suggesting that lncRNAs have a role in disease development. The evaluation of specific transcripts involved the assessment of the tissue specificity index τ (Tau) [54]. The larger mean tau value for lncRNAs indicated that the expression of lncRNAs (0.69) was more stage-specific than the expression of mRNAs (0.56) (Wilcoxon–Mann–Whitney test: *p* = 1.872375e-14) (Fig. 3D).

**Fig. 4:**
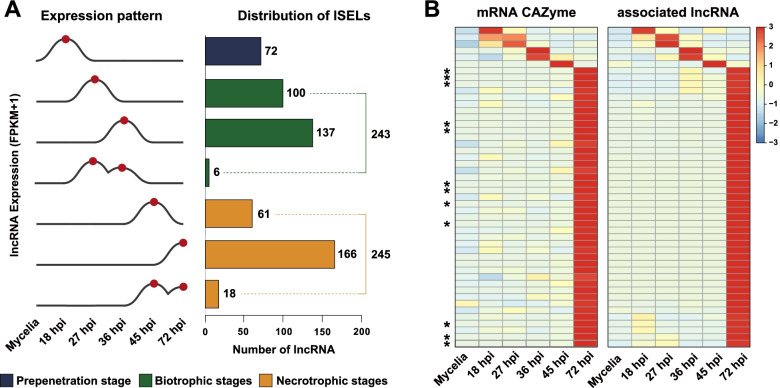
Infection stage-specific lncRNAs and their target genes. **A** Numbers of lncRNAs according to expression pattern. Red spots indicate an expression peak at each stage. **B** Expression heatmap of infection-specifically expressed lncRNAs and their CAZyme target genes. FPKM values were normalized across conditions based on Z-scores. Normalizations of mRNAs and lncRNAs were performed separately. * indicates a functionally characterized xylanase. CAZyme, Carbohydrate-active enzyme

The specificity of lncRNA expression was assessed by categorizing 518 constitutive lncRNAs (tau ≤ 0.5), 1,328 intermediate lncRNAs (0.5 < tau ≤ 0.8), and 755 specific lncRNAs (tau > 0.8) based on the stage specificity index. Of the specific lncRNAs, 195 mycelia-specifically expressed lncRNAs and 560 ISELs were detected. LncRNAs identified during infection included 72 lncRNAs at the pre-penetration stage (18 hpi), 243 lncRNAs at the biotrophic stage (27–36 hpi), and 245 lncRNAs at the necrotrophic stage (45–72 hpi) (Fig. 4A, Additional file 3: Table S1).

### Prediction of stage-specifically expressed lncRNA

The functional roles of lncRNAs were predicted by investigating target genes using two distinct methods. ISELs were the focus of analysis because of their biological importance during infection. In total, 157 protein-coding genes from 143 ISELs were predicted to be cis-targeted genes based on genomic proximity. Trans-targeted genes (242) were predicted from 127 ISELs based on sequence complementarity. Fifty-six ISELs and 34 target genes were found using both methods, resulting in 214 predicted ISELs and 365 predicted target genes. Biological functions were inferred by conducting GO term enrichment analysis. The most enriched GO terms of the target genes groups included “carbohydrate metabolic process” and “interaction with host” terms (Additional file 4: Table S2). The terms “binding" and “mycelium development" were enriched for the target gene set for mycelia-specific lncRNA expression (Additional file 5: Table S3).

Forty-eight of the ISEL-target pairs belonged to carbohydrate-active enzyme (CAZyme) gene families involved in carbohydrate metabolic processes. A positive correlation was found for the majority of pairs (43 of 48), which had the highest expression in the necrotrophic stage (Fig. 4B). ISEL target genes were queried against PHI-base to identify pathogenesis-related genes [53]. As a result, 23 target genes were matched to the gene set from PHI-base (Table 2). The proportion of the pathogenesis-related genes from PHI-base was higher in the target genes of ISELs than those of non-ISELs (two-proportions z-test: *p* = 0.01085) (Additional file 6: Table S4). The majority of these genes were targeted by trans-acting lncRNAs, with one pair acting through both cis- and trans-regulation. The ISEL-associated genes included 5 catabolic metabolism-related genes (4 xylanases and *MoSNF1*), 2 plant avirulence determinants (*MoCDIP4*, *ACE1*), and 1 hydrophobin gene (*MPG1*).

**Table 2:** Target genes of infection specifically-expressed lncRNAs matched to genes from PHI-base

ISEL	Mode of action	Target gene	Description
MSTRG.14853.1	Trans	MoCDIP4	Plant cell death inducer
MSTRG.8963.1	Trans		
MSTRG.14634.1	Trans	ACE1	Polyketide synthase
MSTRG.10882.1	Trans	MoSNF1	AMP-activated protein kinase
MSTRG.5151.2	Cis	MET12	Methylenetetrahydrofolate reductase
MSTRG.12783.1	Trans	MoSOM1	Transcriptional regulator
MSTRG.14270.3	Cis/trans	MoSSK1	Response regulator
MSTRG.1779.3	Cis	MoCOD1	Zn_2_Cys_6_ transcription factor
MSTRG.8913.3	Trans	MoPER1	GPI anchored-related gene
MSTRG.14270.1	Trans	MGG_08331T0	Endo-1,4-beta-xylanase
MSTRG.14270.2	Trans		
MSTRG.14853.1	Trans		
MSTRG.4487.2	Trans		
MSTRG.4487.3	Trans		
MSTRG.8648.1	Trans		
MSTRG.8648.2	Trans		
MSTRG.8648.1	Trans	MGG_08424T0	Endo-1,4-beta-xylanase
MSTRG.8648.2	Trans		
MSTRG.8655.2	Trans		
MSTRG.14853.1	Trans	MPG1	Hydrophobin
MSTRG.8407.2	Trans		
MSTRG.8648.1	Trans		
MSTRG.8648.2	Trans		
MSTRG.14853.1	Trans	MGG_10730T0	Na^+^-ATPase
MSTRG.13745.1	Trans	MoLDS1	Animal peroxidase
MSTRG.2819.1	Trans	SSM2	Non-ribosomal peptide synthetase
MSTRG.12783.1	Trans	MGG_15019T0	Peroxisomal copper amine oxidase
MSTRG.10882.1	Cis	MoRGS4	G-protein signaling regulator
MSTRG.8407.2	Cis	Pmc1	Vacuolar membrane-located Ca^2+^ pump
MSTRG.13998.6	Trans		
MSTRG.1930.1	Trans	MST12	STE-like transcription factor
MSTRG.1930.3	Trans		
MSTRG.8389.1	Cis	XYL1	Endo-1,4-beta-xylanase
MSTRG.13915.1	Trans	XYL-6	Endo-1,4-beta-xylanase
MSTRG.10882.1	Trans	FZC87	Zn_2_Cys_6_ transcription factor
MSTRG.14853.1	Trans	FZC12	Zn_2_Cys_6_ transcription factor
MSTRG.1930.1	Trans	FZC42	Zn_2_Cys_6_ transcription factor
MSTRG.1930.3	Trans		

### Verification of lncRNA production

**Fig. 5:**
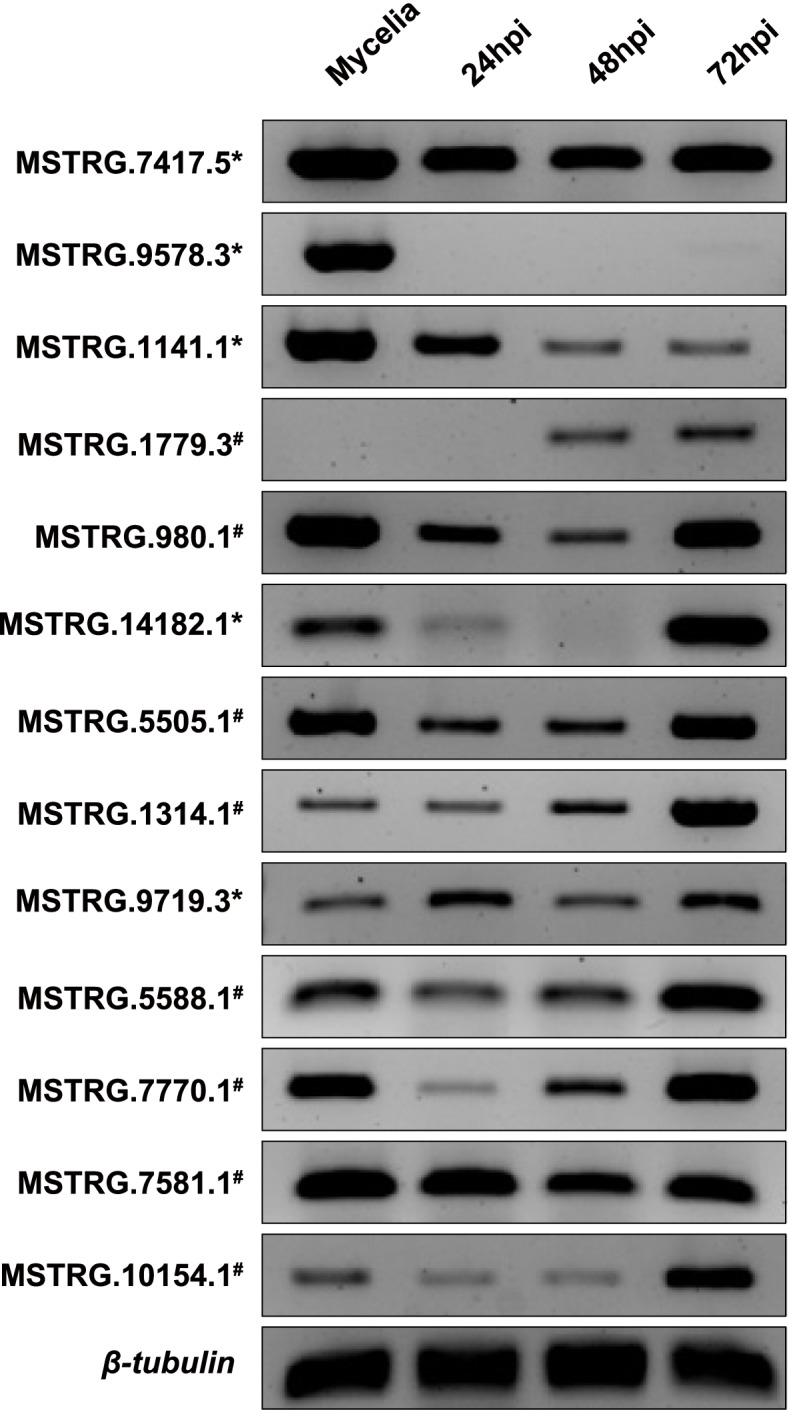
Validation of lncRNA production. Validation of lncRNAs was performed with strand-specific RT-PCR. Templates were cDNAs synthesized from the RNA of mycelia, 24 h post-inoculation (hpi), 48 hpi, and 72 hpi on infected rice leaves. * indicates an antisense lncRNA. # indicates an intergenic lncRNA. *β-tubulin* gene was used as a control. Full-length gels are presented in Fig. S2 (Additional file 5)

LncRNA production was verified using RNA samples from vegetative mycelia and infected rice leaves (Fig. 5, Additional file 7:Fig. S3). The infection process was covered by collecting rice leaves at 24, 48, and 72 hpi for RNA extraction. Five antisense lncRNAs and 8 intergenic lncRNAs were selected for transcript-specific RT-PCR, which can distinguish the exact transcript of interest from overlapping transcripts, including antisense transcripts and alternatively spliced transcripts. All tested lncRNAs were confirmed to be expressed in either the mycelia or during infection.

## Discussion

LncRNAs modulate gene expression at the transcriptional and post-transcriptional levels; they have important roles in various metabolic pathways throughout eukaryotic species [39]. Most lncRNA studies have been performed in model yeasts, while the functional characterization and profiling of plant pathogen lncRNAs have been rarely studied [17, 18]. Genome-wide profiling of plant pathogen lncRNAs in the disease process has been performed in the rice smut fungus *U*. *virens* [30]. The lack of lncRNA studies during disease development limits the understanding of the role of pathogen lncRNAs during infection. In this study, we performed comprehensive profiling of lncRNAs over several infection stages and validated their production (Fig. 5). High-throughput sequencing data yielded 437 million mapped reads, which enabled us to capture non-coding transcripts with low expression levels, as well as transcripts that were actively expressed. While some lncRNAs without a poly(A) tail may have been missed because of poly(A)-capturing library preparation, the impact was presumably minimal because of the large number of lncRNAs transcribed by RNA polymerase II [40]. Specifically expressed transcripts at infection stages would also be underrepresented due to low sequencing depth and ambiguity of strand specificity [41].

*M*. *oryzae* lncRNAs had shorter transcript lengths, fewer exons, lower GC ratios, and temporal-specific expression patterns, suggesting that functional lncRNAs exist in *M*. *oryzae*, because these features were observed in multiple eukaryotic organisms (Fig. 2, Fig. 3) [40]. Low GC content of lncRNAs would be related to their temporal-specific expression and low stability. The positive correlation between the GC content and stability of transcripts was also reported [42]. The roles of lncRNAs are presumed to depend on the protein-coding genes with which they interact. Therefore, the prediction of lncRNA function depends on target gene prediction. Functional characterization of lncRNAs has revealed that both cis- and trans-acting lncRNAs have roles in gene regulation [21, 25]. However, previous fungal lncRNA profiling studies considered only cis-acting lncRNAs [29, 30]. Here, we performed target gene prediction for both cis- and trans-acting lncRNAs; we found more trans-acting lncRNA target genes than cis-acting lncRNA target genes. This extended prediction of target genes enabled us to identify a pool of unbiased lncRNA-associated genes that await further functional characterization of infection-related lncRNAs.

The mean level of lncRNA expression increased for all infection stages, compared with the vegetative growth stage, and a stage-specific pattern was observed. In this study, tau value was used to identify lncRNAs highly expressed only in particular infection stages, providing a well-defined stage-specifically expressed lncRNAs. As expected, we identified more ISELs than mycelia-specifically expressed lncRNAs. Increased expression levels of lncRNAs during the developmental process were also observed in *Fusarium graminearum* sexual reproduction and *U*. *virens* disease development [29, 30]. Our findings and other observations suggest that lncRNAs have roles in the pathogenesis of plant pathogenic fungi.

GO term enrichment analysis revealed that terms related to carbohydrate metabolism were enriched in ISEL-associated genes in *M*. *oryzae* (Additional file 4: Table S2). In *U*. *virens*, transport-related GO terms were enriched during all stages [30]. This difference may be relevant to the distinct lifestyles of biotrophs (*U*. *virens*) and hemibiotrophs (*M*. *oryzae*), although both species infect the same host. PHI-based analysis showed that *M*. *oryzae* lncRNAs may target genes encoding CAZymes, including plant cell wall-degrading enzymes (PCWDEs) (Fig. 4, Table 2). Notably, PCWDEs play important roles in rice blast disease progression by helping to overcome the physical barrier complex composed of cellulose, hemicellulose, pectin, lignin, and xylan [43]. A cellulase-regulating lncRNA was reported in the saprophyte *T*. *reesei*, where cellulases are essential for trophism [20]. Effectors such as *ACE1* and *MoCDIP4* were also found in *M*. *oryzae* lncRNA-associated genes. Effectors secreted from the pathogen act as major virulence determinants [44]. Taken together, the findings thus far suggest that lncRNAs function in the pathogenesis of *M. oryzae* by regulating associated genes.

## Conclusions

In summary, this study reports the first genome-wide lncRNA profile in the model fungal pathogen, *M*. *oryzae*. The profiling of infection-specific lncRNAs and their associated genes suggests that lncRNA may regulate the infection process. Overall, this study provides extensive profiling of lncRNAs and the associated gene repertoire; it also demonstrates the potential roles of lncRNAs involved in rice blast disease development.

## Methods

### RNA extraction and strand‐specific sequencing

*M*. *oryzae* strain KJ201 was obtained from the Center for Fungal Genetic Resources at Seoul National University (Seoul, Korea). Fungal mycelia were cultured with shaking (150 rpm) in a liquid complete medium (0.6% yeast extract, 0.6% tryptone, and 1% sucrose [w/v]) at 25 °C for 3 days. Total RNA was extracted using an Easy-spin total RNA extraction kit (iNtRON Biotechnology, Seoul, Korea), in accordance with the manufacturer’s instructions. Strand-specific cDNA synthesis with NEXTflex Rapid Directional mRNA-seq Kit (Bioo Scientific, Austin, TX, USA) and sequencing were performed at the National Instrumentation Center for Environmental Management at Seoul National University (Seoul, Korea). Shotgun sequencing was used to generate 75.3 million paired-end 151-bp reads using an Illumina HiSeq 2500.

### Collection of *in planta* RNA-seq data

Six *M. oryzae* KJ201 RNA-seq libraries, including different infection stages of rice sheath, were used to identify lncRNA during mycelial growth and disease development (SRA accession no. SRX5076910- SRX5076915) [37]. The RNA-seq data contained paired-end 101-bp reads and included the following stages: vegetative mycelia, pre-penetration stage (18 hpi), biotrophic stage (27 and 36 hpi), and necrotrophic stage (45 and 72 hpi). These stages included appressorium formation (pre-penetration, 18 hpi), penetration and development of primary invasive hyphae (biotrophic stage, 27 hpi), development and growth of invasive hyphae (biotrophic stage, 36 hpi), active growth of invasive hyphae into neighboring host cells (necrotrophic stage, 45 hpi), and extensive proliferation and killing of host cells (necrotrophic stage, 72 hpi).

### Transcriptome assembly

Raw reads were processed to remove low-quality reads and trim adapter sequences using NGS QC Toolkit v2.3.3 [45]. The resulting reads were mapped against the *M. oryzae* reference genome (MG8, Ensembl annotation 29) using HISAT2 v2.0.4 [32, 46]. The transcriptome was assembled using the genome-guided method of StringTie v1.3.3 with de novo annotation [47]. Transcriptome assembly proceeded through two steps. In the first step, the strand-specific RNA-seq data was used. Then, *in planta* RNA-seq data and the updated transcriptome annotation from the first step were used in the second step. We used fragments per kilobase of transcript per million mapped read pairs (FPKM) as the expression value. If the expression value for a transcript was < 1 FPKM at all stages, the transcript was considered to be predicted, but not detected. Detected transcripts were used for subsequent analysis.

### Pipeline for lncRNA identification

We used an established computational pipeline to identify lncRNAs. Transcripts whose spliced sequences are shorter than 200 nucleotides were first filtered out. The assembled transcripts were then compared with protein-coding genes and categorized using Gffcompare [48]. We regarded antisense transcripts (class code “x”), sense transcripts (class codes “j” and “o”), intronic transcripts (class code “i”), and intergenic transcripts (class codes “u” and “p”) as novel transcripts. Known non-coding RNAs (tRNAs, rRNAs, snRNAs, and snoRNAs) were removed using Infernal v1.1.1 based on Rfam database release 14.0 [49, 50]. The coding potentials of transcripts were assessed using CPAT v.1.2.2 [51]. To maximize lncRNA detection, training was performed using transcript sequences of *F*. *graminearum* and the coding potential cutoff was set to 0.54 (Additional file 8: Table S5, Additional file 9: Fig. S4). Transcripts with coding potential below the cutoff were included; transcripts containing any known Pfam domain were removed using InterProScan version 5.29–68.0 [52].

### LncRNA conservation analysis

The 2,601 M. *oryzae* lncRNAs identified in this study were BLAST searched against known lncRNAs downloaded from RNAcentral with an E-value cutoff of 1e-5 [53]. The level of conservation between *M. oryzae* lncRNAs and other Magnaporthales species was assessed by BLAST searching predicted *M*. *oryzae* lncRNAs and annotated mRNAs against the genomes of eight Magnaporthales species (*Magnaporthe grisea*, *Gaeumannomyces graminis*, *Magnaporthe poae*, *Magnaporthiopsis rhizophila*, *Magnaporthiopsis incrustans*, *Magnaporthe salvinii*, *Ophioceras dolichostomum*, *Pseudohalonectria lignicola*), as well as *Neurospora crassa* as an outgroup, with an E-value cutoff of 1e-5. The genomes of *M*. *grisea*, *G*. *graminis*, *M*. *poae*, and *N*. *crassa* were obtained from the Comparative Fungal Genomics Platform (http://cfgp.riceblast.snu.ac.kr) [54]. The genomes of *M*. *rhizophila*, *M*. *incrustans*, *M*. *salvinii*, *O*. *dolichostomum*, and *P*. *lignicola* were downloaded from the National Center for Biotechnology Information [55].

### Assessment of stage specificity and prediction of stage-specific lncRNAs

The stage specificities of transcripts were determined using the tissue specificity index as described previously [56].

$$\uptau =\frac{\sum_{i=1}^{n}(1-\widehat{{x}_{i}})}{n-1};\widehat{{x}_{i}}=\frac{{x}_{i}}{\underset{1\le i\le n}{\mathrm{max}}{x}_{i}}$$

where n is the number of stages and *x*_*i*_ is the expression level at stage i. The index varies from 0 (consistently expressed transcripts) to 1 (perfectly stage-specific transcripts).

Stage-specific lncRNAs were selected based on the following criteria: Tau > 0.8 and (FPKM of the stage with the highest expression)/(FPKM of the stage with the second highest expression) > 2. LncRNAs with expression during the first and second peaks of the biotrophic stages were considered biotrophic stage-specific lncRNAs; lncRNAs with expression during both the first and second peaks of the necrotrophic stages were considered necrotrophic stage-specific lncRNAs.

### Target gene prediction

Protein-coding genes co-expressed with lncRNAs were identified using Pearson correlation coefficients, which were calculated between each mRNA–lncRNA pair based on expression values. Genes with an absolute value of coefficient > 0.9 were considered to be co-expressed. For these genes, possible target genes for cis- or trans-regulation were predicted using two independent criteria. For cis-target gene prediction, genes within a 10-kb window upstream or downstream of the lncRNAs were considered. For trans-target gene prediction, transcript sequence complementarity and RNA duplex energy were used to assess the impact of lncRNA binding on mRNA molecules using RNAplex (parameter: 1e-60) [57]. Target genes were then subjected to Gene Ontology (GO) term enrichment analysis at a 5% false discovery rate using Blast2GO and AgriGO v2.0 [58, 59]. Pathogenesis-related genes were identified by querying target genes against a pathogen-host interactions database (PHI-base) [60].

### Validation of lncRNA transcript production

The validation of lncRNA production was measured on the basis of lncRNA expression during vegetative mycelia and infection stages using strand-specific reverse transcription PCR (RT-PCR). Rice cultivar Nakdong was grown in a growth chamber at 28℃ and 80% humidity with a 16/8-h light/dark photoperiod. Four-week-old rice seedlings were inoculated with *M*. *oryzae* KJ201 conidial suspension with 20 × 10^4^ conidia/mL in 250 ppm Tween 20 using a sprayer. The inoculated plants were incubated for 24 hpi, 48 hpi, and 72 hpi. cDNA was synthesized using ImProm-II™ Reverse Transcription System (Promega, Madison, WI, USA), in accordance with the manufacturer’s instructions. For strand-specific reverse transcription, transcript-specific primers were designed as previously reported [61]. Reverse transcription reactions were carried out with 200 ng of total RNA, 1 μl of 4 pmol/μl of transcript-specific primers, 2 μl of synthesized cDNA, and 1 μl of 10 pmol/μl nested primers, which were designed to amplify only the synthesized cDNA. I-star-max II PCR master mix was added for a total volume per reaction of 10 μl. Primers used in all RT-PCR experiments are listed in Table S6 (Additional file 10).

## Supplementary Information

**Additional file 1: Figure S1. **Venn diagram showing the number of lncRNAs expressed among stages.

**Additional file 2: Figure S2. **Conservation of lncRNAs among Magnaporthales species and *N*. *crassa*.

**Additional file 3: Table S1. **Information of infection stage-specifically expressed lncRNAs.

**Additional file 4: Table S2. **Enriched GO terms of infection stage-specifically expressed lncRNA target genes.

**Additional file 5: Table S3. **Enriched GO terms of mycelia-specifically expressed lncRNA target genes.

**Additional file 6: Table S4. **Contingency table of pathogenesis-related genes matched to PHI-base.

**Additional file 7: Figure S3. **Full-length gel pictures for strand-specific RT-PCR data of each lncRNA expression in Fig. 5.

**Additional file 8: Table S5.** List of transcripts used as CPAT training set.

**Additional file 9:** **Figure S4. **Coding potential model in filamentous fungi.

**Additional file 10: Table S6.** The primers used in this study.

## Data Availability

All the data supporting our findings are contained within the manuscript. All raw transcriptome data reported in this article have been deposited in the Sequence Read Archive (SRA) under accession number SRP332970. The used datasets include: The genome of *M*. *oryzae* in Ensembl (MG8). (https://fungi.ensembl.org/Magnaporthe_oryzae/Info/Annotation/); The genomes of *M*. *grisea*, *G*. *graminis*, *M*. *poae*, and *N*. *crassa* in CFGP (BR29, R3-111a-1, ATCC 64,411, OR74A). (http://cfgp.riceblast.snu.ac.kr); The genomes of *M*. *rhizophila*, *M*. *incrustans*, *M*. *salvinii*, *O*. *dolichostomum*, and *P*. *lignicola* in NCBI Assembly (GCA_003049465.1, GCA_003049425.1, GCA_003049435.1, GCA_003049485.1, GCA_003049395.1). (https://www.ncbi.nlm.nih.gov/assembly/); Rfam 14.0 in Rfam database (https://rfam.xfam.org/); Pfam 31.0 in InterPro (https://www.ebi.ac.uk/interpro/); Raw transcriptome data in NCBI SRA (SRX5076910-SRX5076915). (https://www.ncbi.nlm.nih.gov/sra/); The cDNA, CDS, and ncRNA sequences of *Fusarium graminearum* (RR1). (https://fungi.ensembl.org/Fusarium_graminearum/Info/Index); Public access to all databases is open.

## Abbreviations

LncRNA: Long non-coding RNA
ISEL: Infection-specifically expressed lncRNA
FPKM: Fragments per kilobase of transcript per million mapped read pairs
GO: Gene Ontology
CAZyme: Carbohydrate-active enzyme
PCWDE: Plant cell wall-degrading enzyme
