# Supplementary figures and images for: Genome-wide profiling of long non-coding RNA of the rice blast fungus Magnaporthe oryzae during infection
Source: BMC Genomics. 2022 Feb 15;23:132. doi: 10.1186/s12864-022-08380-4 (PMC8845233; doi:10.1186/s12864-022-08380-4)

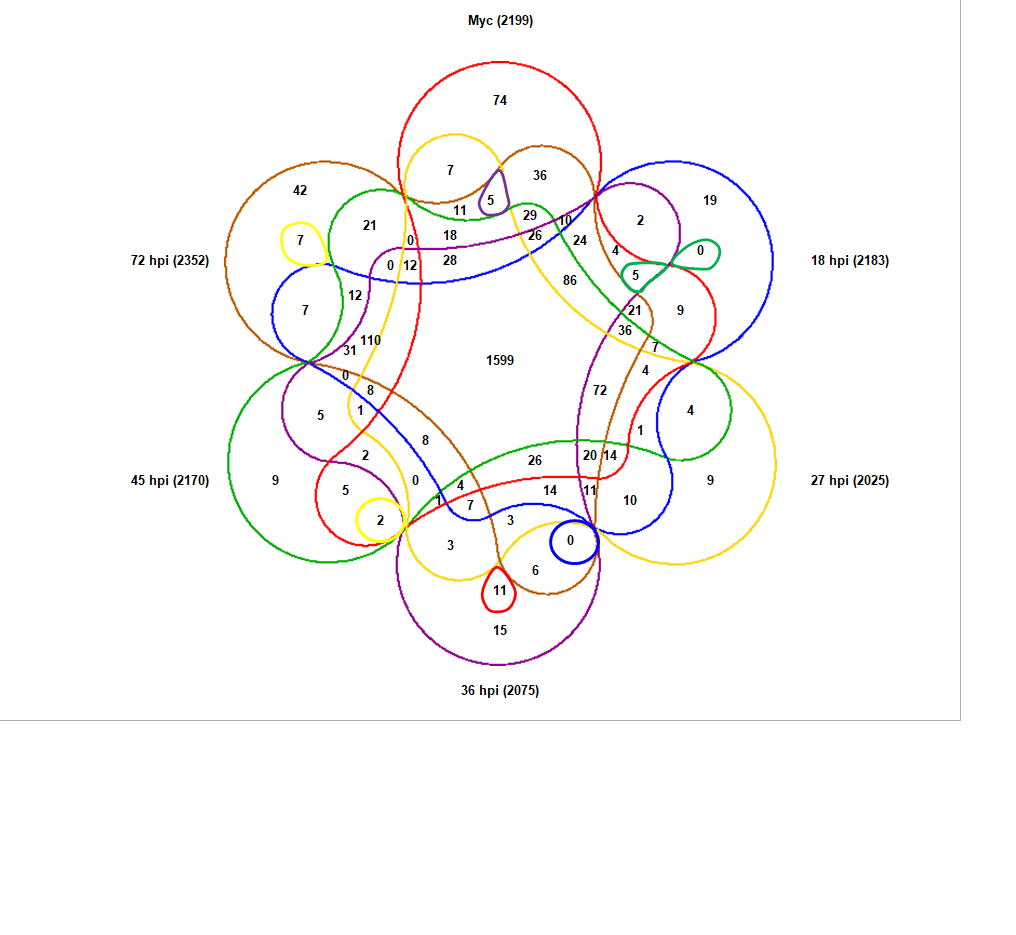


**Figure S1.** Venn diagram showing the number of lncRNAs expressed among stages.

Supplement: Supplementary file 1 — Additional file 1: Figure S1. Venn diagram showing the number of lncRNAs expressed among stages. [file 12864_2022_8380_MOESM1_ESM.docx]
