# Supplementary material for: Genome-wide profiling of long non-coding RNA of the rice blast fungus Magnaporthe oryzae during infection
Source: BMC Genomics. 2022 Feb 15;23:132. doi: 10.1186/s12864-022-08380-4 (PMC8845233; doi:10.1186/s12864-022-08380-4)
Supplement: Supplementary file 2 — Additional file 2: Figure S2. Conservation of lncRNAs among Magnaporthales species and N. crassa. [file 12864_2022_8380_MOESM2_ESM.docx]

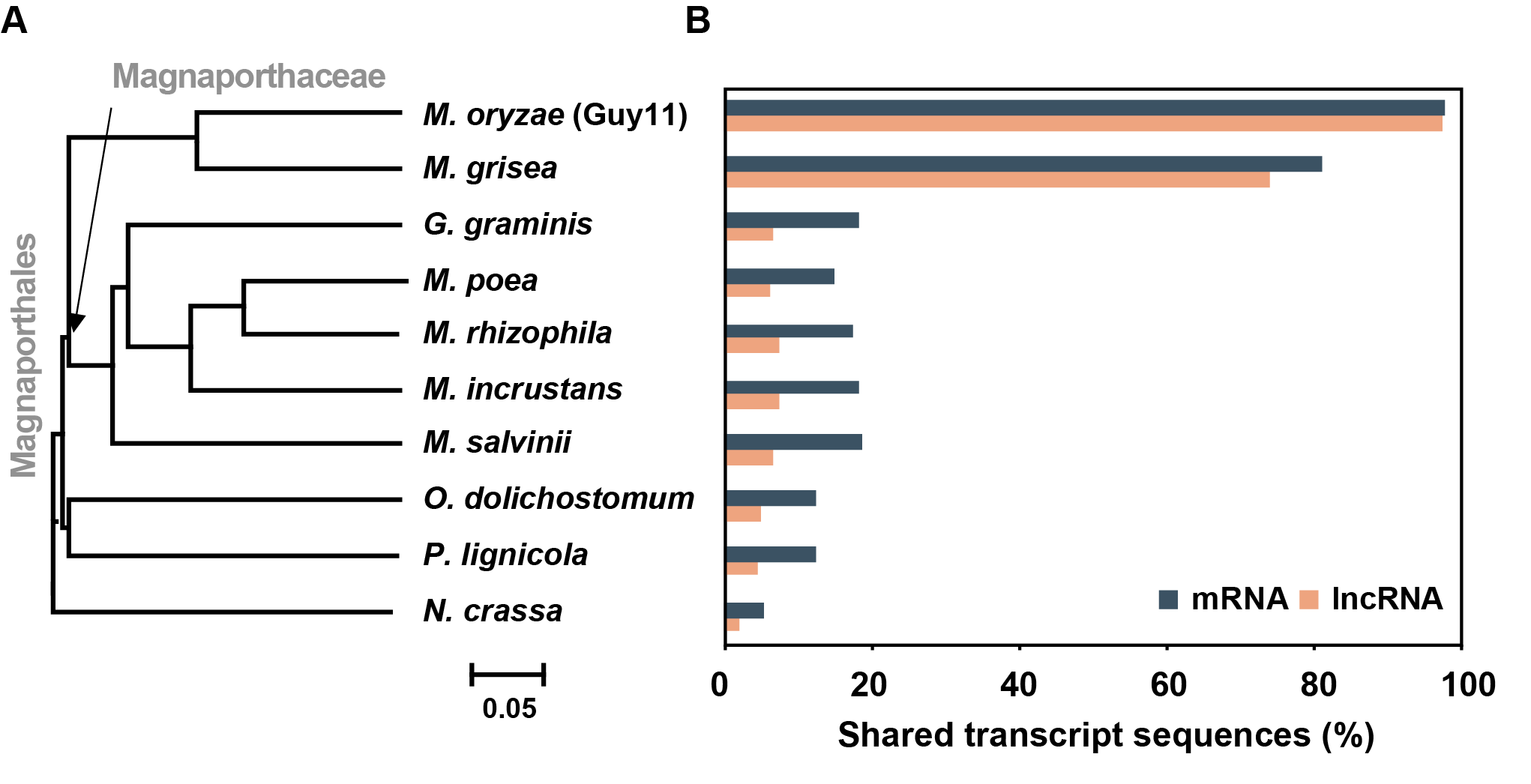


**Figure S2.** Conservation of lncRNAs among Magnaporthales species and *N*. *crassa*.

Proportion of mRNAs (bluish green) and lncRNAs (brown) identified using BLASTn and *M*. *oryzae* lncRNA sequences against the genome with an e-value cutoff < 1e-5. *M*. *oryzae*, *Magnaporthe* *oryzae*; *M*. *grisea*, *Magnaporthe* *grisea*; *G*. *gramininis*, *Gaeumannomyces* *graminis*; *M*. *poae*, *Magnaporthe* *poae*; *M*. *rhizophila*, *Magnaporthiopsis* *rhizophila*; *M*. *incrustans*, *Magnaporthiopsis* *incrustans*; *M*. *salvinii*, *Magnaporthe* *salvinii*; *O*. *dolichostomum*, *Ophioceras* *dolichostomum*; *P*. *lignicola*, *Pseudohalonectria* *lignicola*; *N*. *crassa*, *Neurospora* *crassa*.
