# Supplementary material for: Genome-wide profiling of long non-coding RNA of the rice blast fungus Magnaporthe oryzae during infection
Source: BMC Genomics. 2022 Feb 15;23:132. doi: 10.1186/s12864-022-08380-4 (PMC8845233; doi:10.1186/s12864-022-08380-4)
Supplement: Supplementary file 7 — Additional file 7: Figure S3. Full-length gel pictures for strand-specific RT-PCR data of each lncRNA expression in Fig. 5. [file 12864_2022_8380_MOESM7_ESM.docx]

**A**

**MSTRG_1141.1**

**M**

**24**

**48**

**72**

**MSTRG_14182.1**

**M**

**24**

**48**

**72**

**MSTRG_1779.3**

**M**

**24**

**48**

**72**


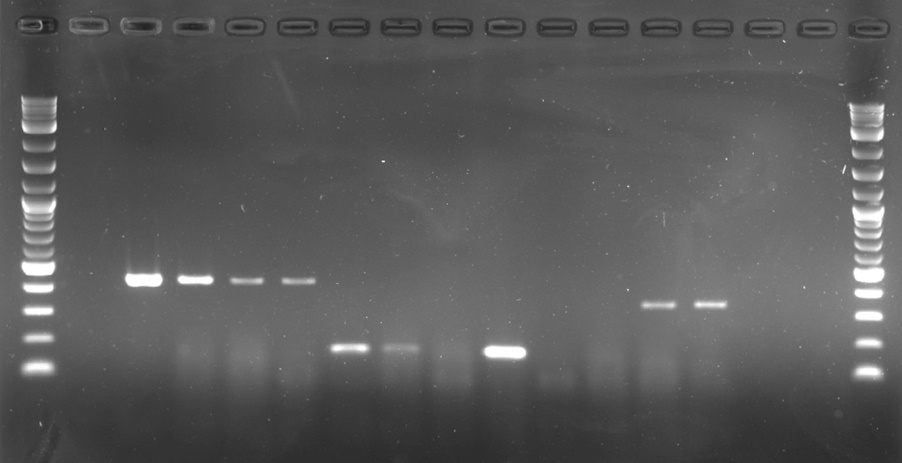


**L**

**L**

**500 bp -**

**400 bp -**

**300 bp -**

**200 bp -**

**100 bp -**

**B**

**M**

**24**

**48**

**72**

**MSTRG_9578.3**

**M**

**24**

**48**

**72**

**MSTR_7417.5**

**M**

**24**

**48**

**72**

**MSTRG_1314.1**


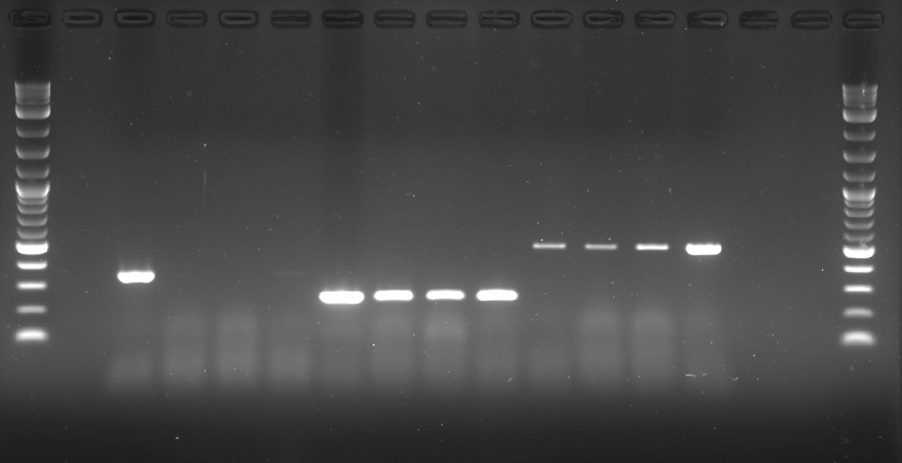


**L**

**L**

**500 bp -**

**400 bp -**

**300 bp -**

**200 bp -**

**100 bp -**

**600 bp -**

**C**

**M**

**24**

**48**

**72**

**MSTRG_5588.1**

**M**

**24**

**48**

**72**

**MSTRG_7770.1**


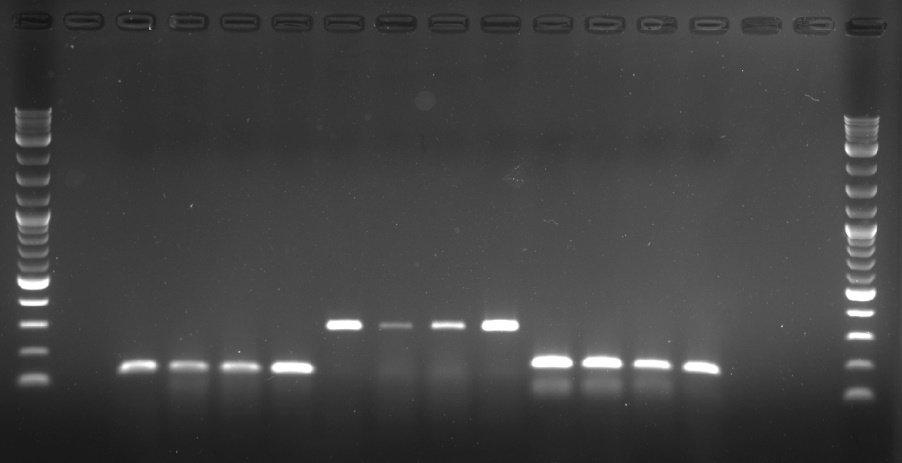


**M**

**24**

**48**

**72**

**MSTRG_7581.1**

**L**

**L**

**400 bp -**

**300 bp -**

**200 bp -**

**100 bp -**

**D**


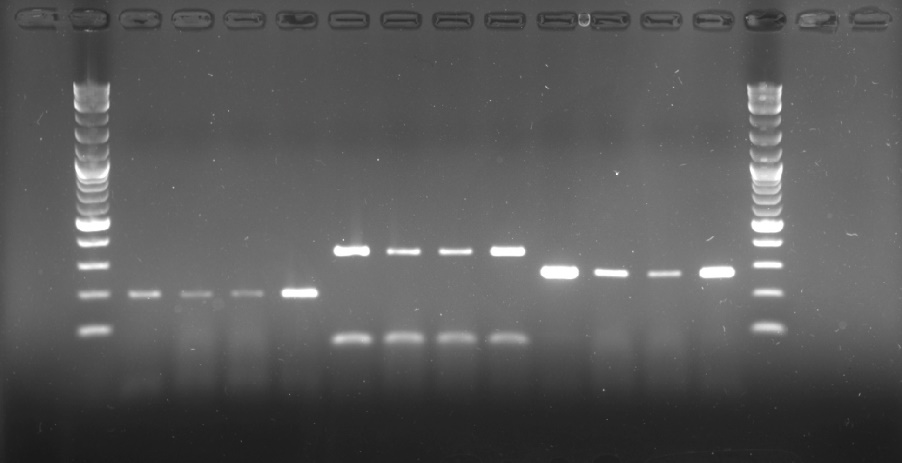


**MSTRG_10154.1**

**M**

**24**

**48**

**72**

**MSTRG_5505.1**

**M**

**24**

**48**

**72**

**MSTRG_980.1**

**M**

**24**

**48**

**72**

**L**

**L**

**400 bp -**

**300 bp -**

**200 bp -**

**100 bp -**

**E**

**M**

**24**

**48**

**72**

**MSTRG_9719.3**

**M**

**24**

**48**

**72**

***β-tubulin***

**L**

**L**

**400 bp -**

**300 bp -**

**200 bp -**

**100 bp -**


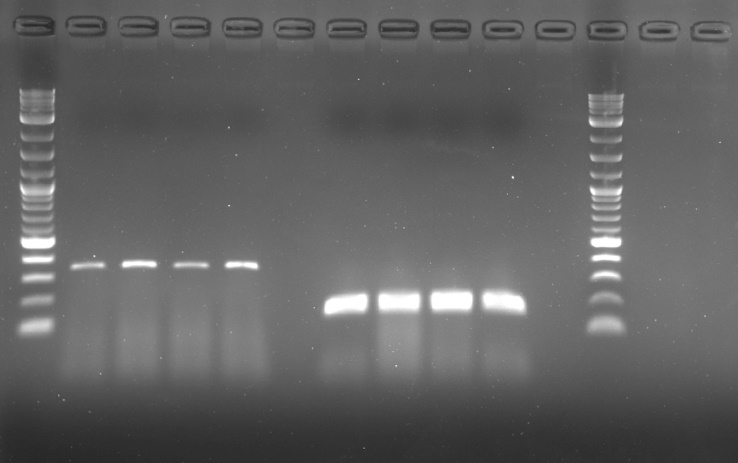


**Figure S3**. Full-length gel pictures for strand-specific RT-PCR data of each lncRNA expression in Fig. 5.
