# Supplementary material for: Genome-wide profiling of long non-coding RNA of the rice blast fungus Magnaporthe oryzae during infection
Source: BMC Genomics. 2022 Feb 15;23:132. doi: 10.1186/s12864-022-08380-4 (PMC8845233; doi:10.1186/s12864-022-08380-4)
Supplement: Supplementary file 9 — Additional file 9: Figure S4. Coding potential model in filamentous fungi. [file 12864_2022_8380_MOESM9_ESM.docx]

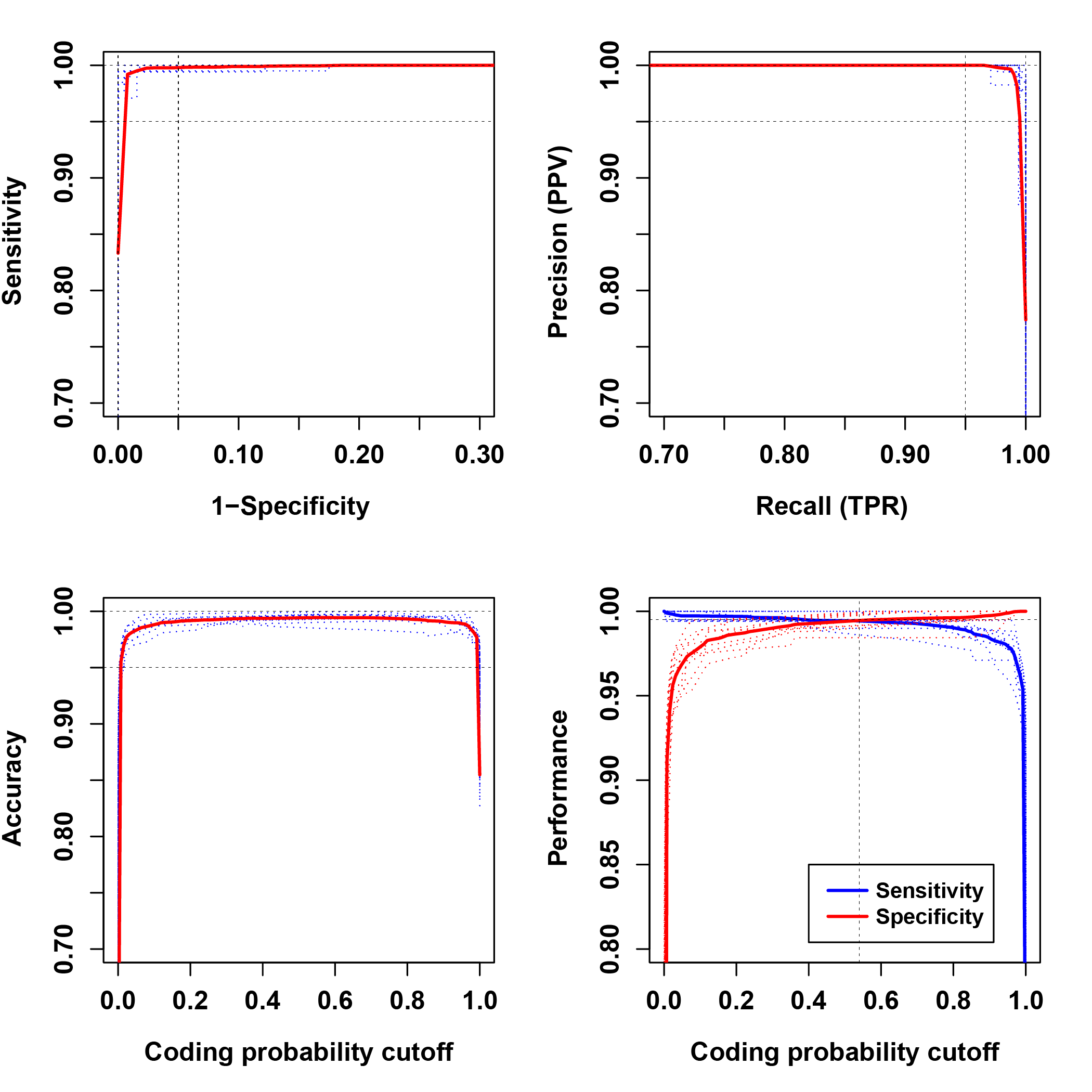


**Figure S4.** Coding potential model in filamentous fungi.

Performance evaluation of non-coding transcript prediction. Two-graph receiver operating characteristic analysis was performed to determine an optimal CPAT cutoff value for non-coding transcript calls. The dashed curves represent 10-fold cross-validation, while solid curves represent the mean curve from 10 validation runs. Sensitivity measures the proportion of positives that are correctly identified. Specificity measures the proportion of negatives that are correctly identified.
